# Facial palsy after orthognathic surgery: An integrative analysis of literature reports and an illustrative case

**DOI:** 10.4317/jced.62082

**Published:** 2024-11-01

**Authors:** Tatiane Fonseca Faro, Joana de Ângelis Alves Silva, Gustavo José de Luna Campos, Maristela Queiroz, Luciana Moraes Studart-Pereira, Caroline Vieira de Lucena, José Rodrigues Laureano Filho

**Affiliations:** 1 DDS, MSc, PhD - Department of Oral and Maxillofacial Surgery, School of Dentistry, University of Pernambuco, Recife, Pernambuco, Brazil; 2 DDS, MSc Student - Department of Oral and Maxillofacial Surgery, School of Dentistry, University of Pernambuco, Recife, Pernambuco, Brazil; 3 DDS, MSc - Private Practitioner, Recife, Pernambuco, Brazil; 4 Physitherapist – Private Practitioner, Recife, Pernambuco, Brazil; 5 Speech Therapist, MSc, PhD, Professor of Departament of Speech Therapy, Federal University of Pernambuco, Recife, Pernambuco, Brazil; 6 Speech Therapist, MSc, DDS Student. Department of Prosthesis and Oral and Facial Surgery, School of Dentistry, Federal University of Pernambuco, Recife, Pernambuco, Brazil; 7 DDS, MSc, PhD, Post Doc Professor, Oral and Maxillofacial Surgeon. Department of Oral and Maxillofacial Surgery, School of Dentistry, University of Pernambuco, Recife, Pernambuco, Brazil

## Abstract

**Background:**

To describe a case of a patient with PFP after orthognathic surgery and discuss cases reports on temporary or permanent facial paralysis, factors that trigger injury, and treatment for facial paralysis associated with orthognathic surgery.

**Material and Methods:**

This study has two parts: a report of the case of a 20- year-old man who underwent orthognathic surgery for facial paralysis, and an integrative literature review on postoperative facial paralysis following the Preferred Reporting Items for Systematic Reviews and Meta-Analyses statements and performed survival analyses of all cases reported to date.

**Results:**

The analysis was composed of 33 patients; 54,5% were male (mean age, 25 years). The right side was most affected by PFP (54.5%). Mandibular (48.5%) and bimaxillary (36.4%) surgeries were the most frequently performed procedures; the mandibular movements ranged from 1 to 18 mm (right side) and 2 to 18 mm (left side). The hypotheses regarding the possible causes of PFP differed between the selected studies. However, compression of the facial nerves was the most common. The use of steroids and physiotherapy were the most described treatment plans. The follow-up period ranged from 1.5 to 36 months (average, 6.12 months), and 78.7% of the patients had complete remission.

**Conclusions:**

Although rare, PFP after orthognathic surgery is one of the most serious complications, as it reduces the quality of life and social interaction of the patients. Therefore, early evaluation should be considered immediately in the postoperative period in patients undergoing orthognathic surgery. There presently is no consensus on the management protocol and establishing a systematization can be beneficial for patients with PFP.

** Key words:**Facial palsy, facial paralysis, sagittal split ramus osteotomy, orthognathic surgery, complication, nerve damage.

## Introduction

Orthognathic surgery is an elective surgical procedure performed for the treatment and correction of congenital diseases and acquired dentofacial deformities (1,2). In patients undergoing this procedure, it is possible to observe an improvement in the stomatognathic functions (breathing, chewing, and phonoarticulation), posture, and esthetics of the lower and middle thirds of the face (1).

Sagittal split osteotomy is a technique commonly used in orthognathic surgery to correct malocclusion, facial asymmetry and deformities involving the mandible, including retrognathism and mandibular prognathism (2-6). This technique is associated with the potential occurrence of complications during intraoperative and postoperative periods of orthognathic surgery (1). Bleeding, damage to teeth and soft tissues, displacement and condylar dysfunction, bad split, nonunion of bone or bone defects, and postoperative infections are the reported as possible complications associated with orthognathic surgery (1,2,4,5). Although rare, with an incidence between 0.17% and 0.75% (5,7) facial nerve injuries that evolve to facial paralysis have been described and included in the group of potential complications in orthognathic surgery (1,3,4,5,8).

Facial nerve injury after orthognathic surgery usually involves the peripheral facial nerve distal to the stylomastoid foramen (1,9). Possible etiologies of this condition can be didactically divided between those of direct causes, such as intraoperative facial nerve compression or injury (complete or incomplete nerve transection), postoperative edema, and change of position in the styloid process, and indirect causes, such as retraction or excessive traction of the posterior tissue in the mandibular branch (1,4,5,7,9).

Peripheral facial paralysis (PFP) is characterized by unilateral weakness or paralysis of the muscles of facial expression, which can cause difficulties during eating, changes in the control of salivary flow, and asymmetry on the face (1). It is considered one of the most serious complications associated with orthognathic surgery, as it can cause severe esthetic, functional, and mental disorders in the patient, affecting their quality of life and social interaction (1,5,6). Therefore, careful clinical treatment by the maxillofacial surgeon is required (5).

Among the methods available and adopted in the management and treatment of patients with PFP, the scientific literature suggests the use of drug therapy with steroids, such as dexamethasone and anti-inflammatory agents, vitamin B12 injections, physical therapy using transdermal nerve stimulation and electroacupuncture, in addition to the use of eye patches, artificial tears, and ophthalmic ointments used for eye protection if there is an inability to close the eyelids caused by facial nerve palsy after orthognathic surgery (4,9).

This study describes an illustrative case of a patient with PFP after orthognathic surgery, the treatment adopted, and its clinical evolution. Moreover, previous cases of temporary or permanent facial paralysis reported in the scientific literature are presented and discussed.

## Material and Methods

-Study Design

This study is divided into two parts. In the first part, we report a case of postoperative facial paralysis due to orthognathic surgery. In the second part, we performed an integrative review of the literature on postoperative facial paralysis following the Preferred Reporting Items for Systematic Reviews and Meta-Analyses statements and performed survival analyses of all cases reported to date.

-Case Report

We report the case of a 20-year-old male patient with PFP after orthognathic surgery, including the treatment adopted and its clinical evolution.

-Integrative Literature Review

Search strategies

A comprehensive search of case reports of facial paralysis after orthognathic surgery was performed in May 2020 without considering the time constraints in the search process. The databases used in this step were MedLine/PubMed and EMBASE. The searches were performed individually by two authors (LFOM and TFF), and the combination of free text and DeCS/MeSH terms used in the search strategies were as follows: “orthognathic surgery” OR “sagittal branch osteotomy” OR “mandibular osteotomy” OR “orthognathic surgical procedures” OR “prognathism” and “Bell’s palsy” OR “facial palsy” OR “facial nerve injuries.”

In addition, studies in the reference list of selected articles were manually analyzed by the same authors to identify additional articles that could have been lost in the initial search.

Inclusion and exclusion criteria

In this study, only case reports or cases involving postoperative facial paralysis were selected. Moreover, only studies written in English with sufficient clinical data were included. There were no constraints on the date of publication. Animal studies, reviews, and articles that did not report relevant data for this study were excluded.

Study selection

Electronic searches in the databases were independently performed by two authors (JÂAS and TFF). After removing duplicate articles, they listed and selected the publications according to their titles and abstracts and assessed their eligibility. Disagreements were analyzed by a third author (JRLF), and a consensus was reached through discussion.

-Data extraction and analysis

The following data were extracted from each included study: authors and year of publication; parents; sex and age of patients; facial paralysis side; surgery performed; mandibular movement; onset of facial paralysis after surgery; causal hypothesis; treatment approaches; motor therapy; complete cure time and follow-up period.

## Results

-Case Report

**Figure 1:**
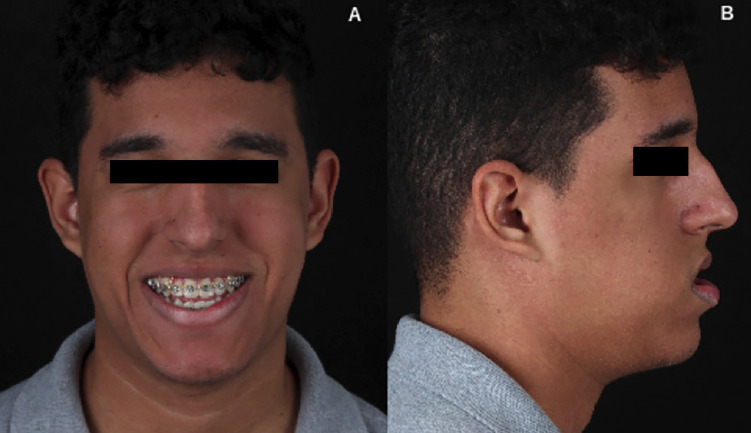
A: Patient with anterior open bite and laterognathia on the left. B: Anteroposterior maxillary deficiency, standard III facial profile.

**Figure 2:**
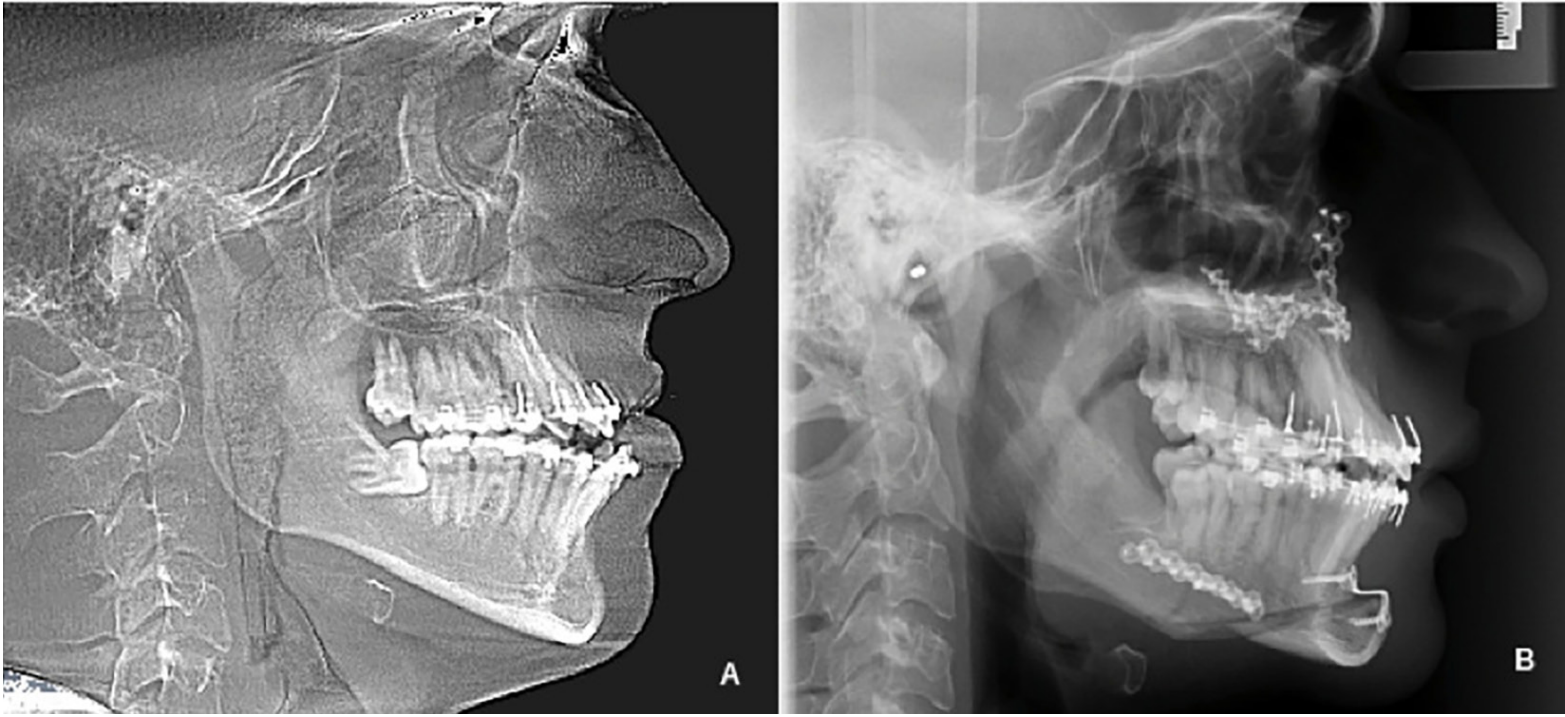
A: Preoperative cephalometry—Skeletal open bite, standard III facial profile. B: Postoperative cephalometry—Open bite correction, standard facial profile I.

**Figure 3:**
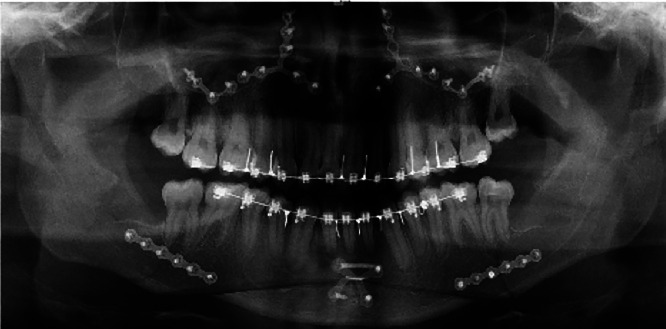
Postoperative panoramic radiograph. The arrows show an area of incomplete traces of the unwanted fracture.

A 20-year-old male patient presented a standard III facial profile with an anterior open bite (Fig. 1). He underwent orthognathic surgery with a 4.5 mm jaw setback, 5.27 mm maxillary advancement, 2 mm roll correction to the left, anterior open bite closure, hourly rotation of the occlusal plane at -7°, and chin advancement to favor the contour of the mentolabial groove (Fig. 2). The third molars were removed at the same time. Regarding the transoperative procedure, it is important to highlight that the sagittal osteotomy of the left mandible evolved with an atypical pattern (Fig. 3), requiring greater detachment of the angle and neck region of the mandibular condyle to improve the observation of the fracture line. Despite the beginning of a bad split, a conventional fixation could be performed. Hospital discharge occurred the day after surgery, following the team’s routine guidance protocols. At that time, no signs of complications were observed.

Immediately after surgery, during the first postoperative control, the following clinical signs of PFP on the left were observed: the presence of global facial asymmetry in the static and dynamic smile, deviation of the buccal rhyme to the right side, partial deviation of the filter to the right side in the formation of the nozzle, deletion of the frontal and nasogenian grooves on the left side, weakness of the eyelid orbicularis muscle showing a mild Bell’s sign in the left eye during eye closure.

PFP is a condition that requires multidisciplinary care. Several specialties may be involved in the rehabilitation of these patients, and the composition of the team, in cases where orthognathic surgeries are part of the initiating factors, is directed by the oral and maxillofacial surgeon.

As soon as the clinical signs of PFP were identified, the team’s conduct was to indicate the beginning of rehabilitation treatment with the accompaniment of the physiotherapy team. Corticosteroids were prescribed. The adopted pharmacological protocol consisted of dexamethasone 4 mg 3x/day (1 day) I.V., prednisolone 20 mg 1×/day (7 days) P.O., prednisolone 10 mg 1x/day (3 days) P.O., B1 Vitamin (100 mg) + B6 Vitamin (100 mg) + B12 Vitamin (5 mg) 3x/day (2 months) P.O., ophthalmic ointment + eye plug.

Rehabilitation therapy with the physiotherapy team started 18 days after surgical intervention and considered the postoperative condition, as well as the sequelae of PFP. Two weekly sessions were held for 12 weeks, totaling 21 sessions. This approach involved manual lymphatic drainage, facial mimic stimulation, myofascial release, kinesiotherapy exercises, and the use of associated resources, such as kinesio taping and laser therapy.

**Figure 4:**
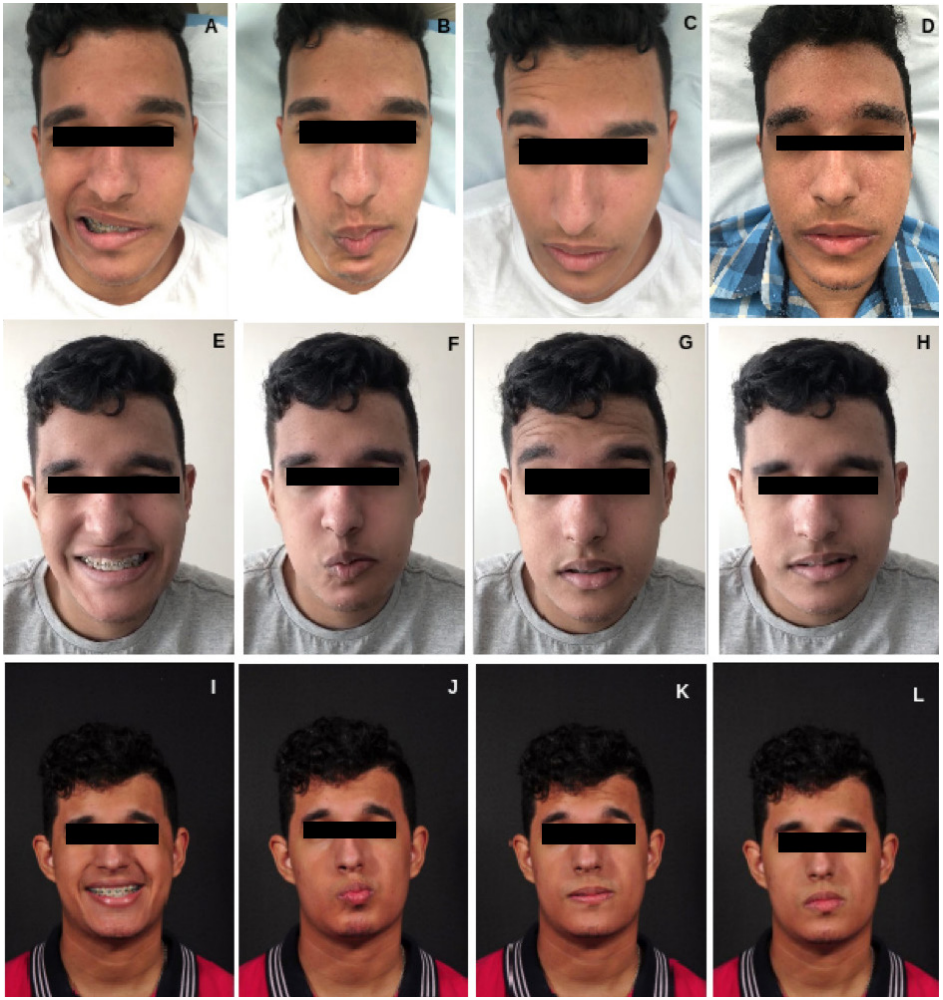
A-D: Facial expression movements performed by the patient before physical therapy where it is possible to observe paralysis of the facial muscles on the left side. E-H: Facial expression movements performed by the patient after the last session of the rehabilitation physical therapy treatment. There is an increase in the movements of the muscles of facial expression on the affected side. I-L: Facial expression movements performed by the patient after a 19-month follow-up. Facial harmony is identified with movement deviations of the lower third of the face.

After intervention and discharge from the physiotherapy team and 106 days after orthognathic surgery, a new evaluation was carried out in which a significant improvement was observed in the condition of movement of the left hemiface muscles (affected by PFP), reduction of asymmetry, and elimination of the Bell’s sign. However, dynamic deviations remain, as in protrusion and lip retrusion, as well as the presence of synkinesis (forced closing of the eyes and lips) (Fig. 4 A-H). Finally, despite the obvious improvement in facial harmony, it was verified in controls (Fig. 4 I-L), the remaining movement deviations that could have been minimized by orofacial myofunctional interventions, such as speech therapy.

-Integrative Review

**Figure 5:**
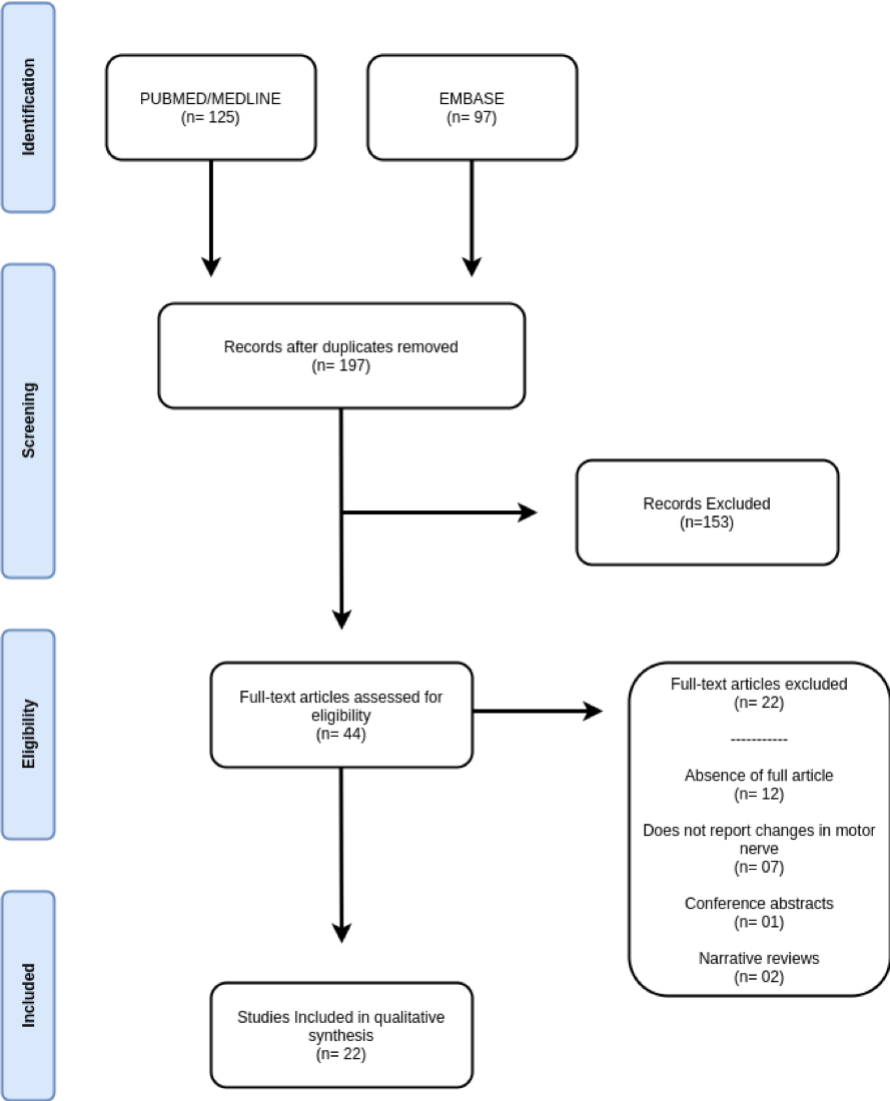
Study selection process.

The search strategy used in this study identified 222 articles from the two databases. After removing 25 duplicate articles (studies cited in more than one database), 197 studies remained. The titles and abstracts of the remaining articles were read by two authors independently, and they selected the articles that were related to PFP after orthognathic surgery. During the reading of titles and abstracts, 153 articles were excluded because they were not related to the topic addressed or did not meet the inclusion criteria for this study. The remaining 44 articles were moved to the full-text reading stage. After this stage, 22 articles were excluded for the following reasons: they were published as conference abstracts or narrative reviews, they did not report changes in the motor nerve, or the full texts were not found. Thus, 22 studies were included in the literature review. Figure 5 summarizes the article selection process from the results obtained from the search strategy adopted for the articles included in the literature review.

The sample of this study consisted of 33 patients. The extracted data are listed in  Table 1. Among these patients, 18 were male (54.5%), and 15 were female (45.5%). The mean age of the patients was 25 years, with minimum and maximum values of 14 and 43 years, respectively. The side most affected by facial peripheral paralysis was the right side (54.5%), which corresponds to 18 patients. Fifteen patients (45.5%) were affected on the left side due to PFP after orthognathic surgery. The surgeries performed were bimaxillary surgery in 12 (36.4%) patients, bimaxillary surgery with chin in three (9.1%) patients, mandibular surgery in 16 (48.5%) patients, and mandibular surgery in two (6.0%) patients. Among the techniques used are bilateral sagittal split ramus osteotomy (BSSRO), bilateral intraoral oblique osteotomy (BIOO), Kole osteotomy, subcondylar osteotomy, chin osteotomy, bimaxillary segmentation, and bimaxillary Wassmund technique. The mandibular movements performed varied from 1 to 18 mm on the right side and 2 to 18 mm on the left side. Facial paralysis occurred shortly after orthognathic surgery in nine patients. In the other patients, the onset of paralysis occurred between 1 and 17 days postoperatively, with an average of 3.75 days.

The hypotheses of the possible causes of PFP include the use of surgical retractors at the depth of wound compression due to the use of retractors during osteotomy, prolonged manipulation of a proximal fragment during osteotomy, a mandibular setback that can cause or along the trunk of the facial nerve, facial nerve damage caused by perforation injury, styloid fracture, control of transoperative bleeding, facial nerve traction, surgical retraction, inflammatory surgical retraction, postoperative edema and/or hematoma substantial compression of the facial nerve, and Bell’s palsy.

Regarding the treatment of PFP after orthognathic surgery, the types of treatment range from pharmacological therapy to motor therapy.

Treatments prescribed for PFP in patients after orthognathic surgery include high doses of steroids, dexamethasone, prednisolone in different dosages, acyclovir 200 g, nimodipine, gamma-aminobutyl acid, adenosine triphosphate, and nicergoline, B complex vitamins (B1, B6, B12), penicillin G, eye drops, and ophthalmic ointments, in addition to the indication in some cases of eye buffer. Motor therapy was adopted in 19 out of 33 cases. Techniques used include physiotherapy, transdermal nerve stimulation, electromyostimulation, electroacupuncture, low-level laser therapy, ultrasound, and exercises provided at home.

The follow-up period ranged from 1.5 to 36 months, with an average of 6.12 months. Two studies did not report the patient’s follow-up time after treatment. Regarding the remission of the signs and symptoms of PFP, 78.7% of the patients (n = 26) had complete dissipation, while seven patients (21.2%) had at least one sequel of facial paralysis.

## Discussion

Orthognathic surgery is a surgical procedure performed to reposition the bones of the maxilla and mandible. This technique is used in the treatment of skeletal and dentofacial deformities, thus promoting the resolution of functional and esthetic problems of the face (1). It is a surgical procedure with a low rate of complications (16).

Facial nerve palsy after orthognathic surgery is described as a rare complication (1,6) with a reported incidence ranging from 0.17% to 0.75% (6,16). Shimada *et al*.’s study that analyzed patients with facial paralysis after orthognathic surgery did not find a prevalence of any sex, age, or side affected by facial paralysis (3).

The facial nerve leaves the base of the skull through the stylomastoid foramen. The main trunk of the VII pair of cranial nerves enters the parotid gland and divides into two branches: upper and lower. The upper branch is divided into the temporal, zygomatic, and upper vestibular branches. The inferior branch is divided into the inferior vestibular, mandibular, and cervical regions. Thus, during the sagittal division procedure, with the patient’s mouth open, there is a distance of less than 1 cm between the posterior edge of the ascending branch of the mandible and the facial nerve (17). Due to this proximity, Vries *et al*. (1993) and Lanigan *et al*. (2004) reported that the close anatomical proximity between the field of operation and the position of the facial nerve may contribute to the occurrence of facial paralysis as a complication of orthognathic surgery (13,17).

It is crucial to differentiate facial paralysis from the central to the peripheral type and determine whether the paralysis is present immediately after surgery or the onset is sometime later, as such information can provide possible indications about the type of damage to the nerve and the location of the lesion (11,13,17). In addition to helping to establish a prognosis, recovery of normal facial nerve function over time (13). In central facial paralysis, there is partial paralysis because contralateral to the lesion, mimicking muscles of the third lower portion of the face are involved, whereas the orbicular muscle of the eye, eyebrow corrugator, and frontal muscles that receive bilateral cortical fibers continue to work, even at a reduced activity. PFP can be divided into two subtypes: intrapedrosal and extrapetrosal. In extrapetrosal lesions, there is total paralysis with loss of voluntary movement of all muscles of the facial mimicry ipsilateral to the lesion. In peripheral paralysis with injury to the intrapetral region, there is also the presence of hearing loss and decreased taste (6,11,12) The absence of PFP immediately after the surgical procedure suggests that the continuity of the nerve remains intact, and direct transection of the nerve bundle is unlikely (9,11,13,17).

There is no consensus on the etiology of facial nerve injury after orthognathic surgery in the literature (3); therefore, the causal hypotheses of facial paralysis are varied. Bisatto *et al*. and Hsu *et al*. pointed out that facial nerve compression was the most likely etiology (1,9). Dendy *et al*. were the first to address the etiological basis for facial nerve injury after sagittal split osteotomy and suggested that the facial nerve can be compressed against the mastoid for three hypothetical reasons: introduction of retractors behind the ascending branch, fracture of the styloid process and its subsequent posterior displacement, or dislocation of the distal segment of the mandible back. The introduction of retractors behind the ascending branch of the mandible is another hypothesis (22). Shimada *et al*. and Rai *et al*. also reported that edema or postoperative hematoma that cause compression of the nerve trunk, anatomical variation of the facial nerve course, ischemia injury caused by vasoconstrictor injection, damage to the facial nerve during the placement of retractors, and use of osteotomes, slip of the drill into the soft tissue, condylar fractures, and unusual fractures as other possible etiologies of facial paralysis after orthognathic surgery (3,6,9,11,12,15). Although facial paralysis occurs after bimaxillary surgery with mandibular advancement, mandibular retreatment procedures are most frequently indicated to present facial paralysis as a complication after orthognathic surgery (3,11,16).

The management of facial paralysis treatment after orthognathic surgery and the etiology of this complication remains debaTable (11) as there is no established therapeutic protocol. The efficacy of steroid therapy to increase nerve regeneration and decrease intraneural pressure and edema is well understood in the literature (6,9,11,16). The early initiation of steroid treatment provides a significant improvement in the condition of the patient,6 and when administered with B complex vitamins (B12), they promote a synergistic association (6,11). Regarding the dosage, the literature points out different dose schedules; some studies claim that high doses of steroids are necessary for the treatment of this complication (16). Rai *et al*., stated that it is essential that steroids are administered preoperatively and during surgery for procedures associated with possible facial nerve injuries. In addition to the use of steroids, Bisatto *et al*. indicated pharmacological treatment with antibiotics and antivirals when PFP is caused by infection. Other substances used and reported in the literature include nimodipine, gamma-aminobutyl acid, adenosine triphosphate, and nicergoline, B complex vitamins (B1, B6, B12), penicillin G, eye drops, and ophthalmic ointments (3,4,6,7,9,14,17).

The treatment of PFP is multidisciplinary and involves several professionals, such as doctors, maxillofacial surgeons, physiotherapists, and speech therapists (1). The use of electrical stimulation has been associated with positive results. Biofeedback, ultrasound therapy, and acupuncture have been associated with motor therapy treatment (6). Connective tissue massage has been used as an adjunct to motor therapy to decrease pain and increase the microcirculation of tissues distant from the treatment area, increasing the plasma concentration of endorphins (9).

In addition to the treatments found in the literature, it is important to emphasize the importance of performing an accurate and in-depth clinical evaluation with standardized instruments that provide information on the degree of involvement, clinical evolution, and prognosis of peripheral facial palsy in patients. A well-conducted assessment offers a more complete view, thus allowing not only the diagnosis of motor changes (tone and mobility) but also the performance of a functional diagnosis, in addition to comparing the evolution of the condition throughout the treatment (23).

Early clinical and neurophysiological investigation of the face of a patient with facial paralysis after orthognathic surgery is important (17) to understand the degree of the injury and its implications for the recovery of the patient (6). The average period for complete recovery of muscle motility is 2-3 months; however, some studies indicate a shorter (3-4 weeks) or longer (4-12 months) recovery period (1,7,12). Shimada *et al*. reported that the period of full recovery of functions was 10 months. Cases of permanent disability are rare (2).

## Figures and Tables

**Table 1:** The extracted data.

Author (Year) country	Age (years)	Sex	Facial palsy side	Surgery performed	A-P mandibular movement	Onset of facial palsy after surgery (days)	Causal hypothesis of facial palsy	Treatment for facial palsy	Motor therapy	Complete healing	Follow-up (months)
Bisatto et al. (2020) (1) Brazil	25	F	Left	Bimaxillary BSSRO	Advancement 7 mm	1	Substantial postoperative swelling, compression due to the use of retractors during the osteotomy, or compression during the BSSO opening.	Prednisolone 20 mg (two capsules in the morning for 5 days, then one capsule in the morning for the next 5 days) Acyclovir 200 g (2 g per day for 5 days)	Physiotherapy	Yes	11
Shimada et al. (2019) (3) Japan	42	M	Right	BSSRO	Setback Right: 6 mm Left: 2 mm	1	Surgical retraction postoperative swelling caused by facial nerve compression	Prednisolone 5 mg (2-11 days) P.O. Eyewash	NR	Yes	2
Maquet et al. (2019) (8) France	14	F	Left	Bimaxillary	NR	After surgery	Surgical retraction Inflammatory/hypoperfusion worsens nerve damage	Corticoid 60 mg (8 days) P.O.	Physiotherapy	No	12
Lee, Lee (2017) (4) Korea	24	F	Right	BSSRO	NR	2	Postoperative hematoma and swelling caused facial nerve compression	Prednisolone 15 mg 8/8 h (4 weeks) P.O. Prednisolone 10 mg (4 weeks) P.O. Prednisolone 5 mg (3 weeks) P.O. Nimodipine 30 mg 8/8 h (4 weeks) P.O.	Physiotherapy + transdermal nerve stimulation + electroacupuncture	Yes	2,5
Hsu et al. (2012) (9) Taiwan	28	M	Left	BIOOs	Setback Right: 6 mm Left: 6 mm	After surgery	NR	Prednisolone 1 mg/kg/day (3 days) P.O. Prednisolone 15 mg/day (5 days) P.O. Vitamin B complex 3x per day (14 days) P.O.	Physiotherapy + lower-level laser therapy	Yes	2,5
Koh et al. (2011) (5) Korea	26	M	Right	Bimaxillary (BSSRO)	NR	4	NR	High dose of steroids	Physiotherapy + electroacupuncture.	Yes	3
	20	M	Right	Bimaxillary (BSSRO)	Setback Right: 18 mm Left: 18 mm	After surgery	The mandibular setback may have caused facial nerve trunk stretching	High dose of steroids	Physiotherapy	No	3
	25	M	Right	BSSRO	Setback Right: 12 mm Left: 10 mm	After surgery	Facial nerve compression by swelling	High dose of steroids	Physiotherapy	Yes	4
Pacheco, Chaurand. (2011) (6) Mexico	22	M	Right	BSSRO (Hunsuc) + Chin	Setback Right: 6 mm Left: 6 mm	3	NR	Dexamethasone 8 mg 4x per day (1 day) I.V. Prednisolone 5mg 4x per day (3 days) P.O. Prednisolone 5 mg 3x per day (3 days) P.O. Prednisolone 5 mg 2x per day (3 days) P.O. Prednisolone 5 mg 1x per day P.O. Vitamin B1 (75 mg), B6 (75 mg) e B12 (0.75 mg) 1x per day (2 months) P.O. Ophthalmic ointment + eye plug	Physiotherapy	Yes	6
Kim et al. (2011) (2) Korea	30	M	Right	Bimaxillary (BSSRO)	Setback Autorotation	After surgery	NR	High dose of prednisolone P.O.	Physiotherapy Electromyostimulation	No	24
Chrcanovic, Custodio (2011)10 Brazil	28	F	Right	Bimaxillary (BSSRO)	NR	2	Facial nerve compression	Corticosteroids	NR	Yes	NR
Choi et al. (2010) (7) Taiwan	20	M	Right	Bimaxillary (BSSRO)	Setback Right: 7 mm Left: 7 mm	3	NR	Corticosteroids	Physiotherapy	Yes	6
	26	F	Left	BSSRO + Kole Osteotomy	Setback Right: 7 mm Left: 10 mm	12	NR	Medical treatment was not specified	Physiotherapy	No	NR
	38	F	Left	Bimaxillary Wassmund + BSSRO + Chin	Advancement 5 mm Advancement 7 mm	17	Bell Palsy	Vit B12 e Gamma-aminobutyl Acid	NR	Yes	5
	25	M	Left	Bimaxillary (BSSRO)	Setback Right: 18 mm Left: 12 mm	6	NR	Unspecified drug treatment	Physiotherapy + transdermal nerve stimulation	Yes	3
	29	F	Left	Bimaxillary + Chin (BSSRO)	Advancement	1	NR	Corticosteroids	NR	Yes	6
	21	M	Right	Bimaxillary Wassmund + BSSRO	Setback	3	NR	Corticosteroids	Physiotherapy + transdermal nerve stimulation	Yes	4
Rai et al. (2008) (11) India	21	F	Left	BSSRO	Setback Right: 4.5 mm Left: 5 mm	3	NR	Dexamethasone 8 mg 4x per day (1 day) I.V. Prednisolone 5 mg 4x per day (3 days) P.O. Prednisolone 5 mg 2x per day (3 days) Ophthalmic ointment	Physiotherapy	Yes	3
Sammartino et al. (2005) (12) Italy	22	F	Right	Bimaxillary (BSSRO)	Setback 6 mm Right Midline correction 4 mm	2	NR	NR	NR	Yes	2.5
	23	F	Right	Bimaxillary (BSSRO)	Setback 3 mm	2	NR	NR	NR	Yes	1.5
Linigan, Hohn. (2004) (13) Canada	43	M	Left	BSSRO	Advancement Autorotation	After surgery	Prolonged manipulation of the proximal fragment during osteotomy Facial nerve stretching	Corticosteroids for a long time	Physiotherapy	No	36
	21	M	Left	BSSRO	Advancement	3	Control of transoperative bleeding caused by facial nerve traction or postoperative hematoma caused facial nerve compression	NR	NR	Yes	2.5
Baek, Song (2004) (14) Korea	24	M	Left	BSSRO	Setback Right: 8 mm Left: 15 mm	16	Bell Palsy	Dexamethasone (1ª week) I.V. Oral prednisolone + nicergoline + adenosine triphosphate (2ª weeks) Adenosine triphosphate + nicergoline (3ª weeks)	Physiotherapy and electrostimulation	Yes	1.5
	20	F	Left	BSSRO	Setback Right: 11 mm Left: 12 mm	After surgery	The mandibular setback may have caused facial nerve trunk stretching	Corticosteroid (5 days) I.V. Prednisolone (40 days) P.O.	Physiotherapy + ultrasound + transdermal nerve stimulation	No	3
Matamedi (1997) (15) Iran	20	F	Right	Subcondylar osteotomy	NR	After surgery	Surgical retraction	Dexamethasone mg 6/6 h (1 week)	Physiotherapy	Yes	3
Sakashita et al. (1996) (16) Japan	21	M	Right	BSSRO	Setback Right: 1 mm Left: 2,5 mm	2	NR	Vitamin B1 (75 mg), B6 (75 mg) e B12 (0.75 mg) 1x per day (2 months) P.O.	Physiotherapy	Yes	3
de Vries et al. (1993) (17) Holand	26	F	Right	BSSRO	NR	1	Facial nerve compression and stretching caused by postoperative swelling	Penicillin G (24 h) Prednisolone (3 days)	NR	No	9
	20	M	Left	BSSRO	Setback 10 mm	1	NR	Eye plug at night	NR	Yes	7
Consolo, Salgarelli (1992) (18) Italy	19	M	Left	Bimaxillary (BSSRO)	Setback 6 mm	2	Use of surgical retractor in depth of the wound	NR	Functional exercises at home	Yes	1.5
Stajcic, Roncevic (1990) (19) Yoguslavia	34	F	Left	Bimaxillary (segmentation) + BSSRO + Chin	Setback 5 mm	After surgery	Facial nerve damage may be caused by drill injury The mandibular setback may have caused facial nerve trunk stretching Control of transoperative bleeding can cause facial nerve traction	NR	NR	Yes	3.5
Zafarulla (1985) (20) England	18	F	Right	OBSM + Chin	Setback 15 mm	1	The mandibular setback may have caused facial nerve trunk stretching and was not caused by postoperative swelling (small/moderate)	NR	NR	Yes	6
Piechuch, Lewis (1982) (21) EUA	27	M	Right	OBSM	NR	1	NR	Corticosteroid (3 days) Multicelulose eye drops + eye plug	NR	Yes	2.75
Dendy (1973) (22) England	23	M	Right	OBSM	Setback	1	The mandibular setback may have caused facial nerve trunk stretching Facial palsy caused by Surgical retraction Styloid fracture	NR	NR	Yes	4

F – Female, M – Male, NR – Not reported, Abbreviations: BIOO, bilateral intraoral oblique osteotomy; BSSRO, bilateral sagittal split ramus osteotomy; OBSM, bilateral sagittal split ramus osteotomy, I.V. – Intravenous, P.O – Per oral

## Data Availability

The datasets used and/or analyzed during the current study are available from the corresponding author.

## References

[1] Bisatto NV, Andriola FO, Barreiro BOB, Maahs TP, Pagnoncelli RM, Fritscher GG (2020). Facial Nerve Palsy Associated With Orthognathic Surgery. The Journal of craniofacial surgery.

[2] Kim YS, Oh ES, Hong JW, Roh TS, Rah DK, Paik HC (2011). Descending necrotizing mediastinitis and facial palsy as serial complications in orthognathic surgery. Journal of Craniofacial Surgery.

[3] Shimada Y, Kawasaki Y, Maruoka Y (2019). Peripheral facial palsy after bilateral sagittal split ramus osteotomy: case report. British Journal of Oral and Maxillofacial Surgery.

[4] Lee JH, Lee KA (2017). New Treatment in Facial Nerve Palsy Caused by Sagittal Split Ramus Osteotomy of Mandible. Arch Craniofac Surg.

[5] Koh KM, Yang JY, Leem DH, Baek JA, Ko SO, Shin HK (2011). Facial nerve palsy after sagittal split ramus osteotomy: follow up with electrodiagnos- tic tests. J Korean Assoc Maxillofac Plast Reconstr Surg.

[6] Pacheco Ruiz L, Chaurand Lara J (2011). Facial nerve palsy following bilateral sagittal split ramus osteotomy for setback of the mandible. International Journal of Oral and Maxillofacial Surgery.

[7] Choi BK, Goh RC, Chen PK, Chuang DC, Lo LJ, Chen YR (2010). Facial nerve palsy after sagittal split ramus osteotomy of the mandible: mechanism and outcomes. J Oral Maxillofac Surg.

[8] Maquet C, Evrard M, Kerbrat JB, Bastien AV, Adnot J, Trost O (2020). A case of severe facial palsy following bimaxillary osteotomy: It is time to update the pre- surgery patient fact sheet. Journal of stomatology, oral and maxillofacial surgery.

[9] Hsu HA, Chang YC, Lee SP, Chen YW (2012). Myofascial pain syndrome may interfere with recovery of facial nerve palsy after orthognathic surgery - A case report. Journal of Oral and Maxillofacial Surgery.

[10] Chrcanovic BR, Custodio AL (2011). Optic, oculomotor, abducens, and facial nerve palsies after combined maxillary and mandibular osteotomy: case report. J Oral Maxillofac Surg.

[11] Rai KK, Shivakumar HR, Sonar MD (2008). Transient facial nerve palsy following bilateral sagittal split ramus osteotomy for setback of the mandible: a review of incidence and management. J Oral Maxillofac Surg.

[12] Sammartino G, Califano L, Grassi R, Liccardo F, Marenzi G, Grivetto F (2005). Transient facial nerve paralysis after mandibular sagittal osteotomy. Journal of Craniofacial Surgery.

[13] Lanigan DT, Hohn FI (2004). Facial nerve injuries after sagittal split mandibular ramus osteotomies for advancement: a report of 2 cases and review of the literature. J Oral Maxillofac Surg.

[14] Baek RM, Song YT (2004). Transient total facial palsy after bilateral sagittal split ramus osteotomy. Plast Reconstr Surg.

[15] Motamedi MH (1997). Transient temporal nerve paresis after intraoral subcondylar ramus osteotomy. J Oral Maxillofac Surg.

[16] Sakashita H, Miyata M, Miyamoto H, Miyaji Y (1996). Peripheral facial palsy after sagittal split ramus osteotomy for setback of the mandible. A case report. Int J Oral Maxillofac Surg.

[17] de Vries K, Devriese PP, Hovinga J, van den Akker HP (1993). Facial palsy after sagittal split osteotomies. A survey of 1747 sagittal split osteotomies. J Craniomaxillofac Surg.

[18] Consolo U, Salgarelli A (1992). Transient facial nerve palsy following orthognathic surgery: A case report. Journal of Oral and Maxillofacial Surgery.

[19] Stajcic Z, Roncevic R (1990). Facial nerve palsy following combined maxillary and mandibular osteotomy. Journal of Cranio-Maxillo-Facial Surgery.

[20] Zafarulla MY (1985). Iatrogenic facial nerve injury. Int J Oral Surg.

[21] Piecuch JF, Lewis RA (1982). Facial nerve injury as a complication of sagittal split ramus osteotomy. J Oral Maxillofac Surg.

[22] Dendy RA (1973). Facial nerve paralysis following sagittal split mandibular osteotomy: a case report. British Journal of Oral Surgery.

[23] Pereira MM, Bianchini EMG, Silva MFF, Palladino RRR (2021). Speech-language-hearing instruments to assess peripheral facial palsy: an integrative literature review. Revista CEFAC.

